# A comparison of an electron diffraction and an X-ray diffraction experiment from a single protein microcrystal lamella

**DOI:** 10.1063/4.0000850

**Published:** 2025-10-27

**Authors:** Adam D Crawshaw, David Owen, Miss Melissa R Whyte-Fink, Anna J Warren, Pedro Nunes, Jose Trincao, Alistair Siebert, Gwyndaf Evans

**Affiliations:** 1 Diamond Light Source, Didcot, Oxfordshire, United Kingdom; 2 University of St. Andrews, St Andrews, Fife, United Kingdom; 3 Rosalind Franklin Institute, Didcot, Oxfordshire, United Kingdom

## Abstract

X-ray diffraction (XRD) of microcrystals is signal-to-noise limited by the inherently weak diffraction. As such, Electron diffraction (ED) is increasingly used to measure diffraction data from submicron crystals, or those deemed too small for XRD due the stronger interaction of electrons with matter. However, many samples which are too thin for XRD are often too thick for ED using the currently available electron beam energies (<300 keV) and hence require thinning by focussed ion beam milling (FIB) which adds additional sample preparation steps. In addition to determining structures from nanocrystals, ED provides Coulomb potential data which are complementary to that obtained with XRD. As such ED data may be necessary to answer particular scientific questions.

The macromolecular crystallography beamline, VMXm, at Diamond Light Source, has been optimised for maximising the S:N in XRD experiments with a variable focus high-energy (>20 KeV) X-ray beam, with in-vacuum endstation and the use of low background cryoTEM grids for crystal mounting [1], [2]. This has allowed VMXm to collect high-resolution rotation data from single crystals measuring ∼1.2 μm which were only previously tractable using an X-ray Free Electron Laser [3]. This has pushed the amenable sample envelope at synchrotrons to new dimensions and perhaps near to the practical limit of XRD. Indeed, simulations have predicted the limit to be ∼0.5 μm thick in the case of lysozyme, assuming photoelectron escape [4]. This XRD beamline opens up the possibilities to directly compare XRD and ED datasets and understand the complementarity of these experiments.

In this work we present data from cubic human insulin crystals that have been thinned by FIB milling from ∼10 μm to various submicron thicknesses. 200 kV ED data were then collected from these lamellae before XRD data were measured from the same lamellae using VMXm. It was possible to obtain a complete XRD dataset to 2.45 Å using a 1.68 μm^3^ illuminated volume and a 2.04 Å ED dataset from the same 0.25 μm lamella. We have demonstrated that the data quality is comparable between ED and VMXm from the same crystal, while giving an opportunity to directly compare X-ray and electron derived maps. This includes the comparison of the radiation damage each experiment imparts on the sample [5] as well as the information content [6]. This work indicates that the usable sample envelope for synchrotron X-rays extends to much thinner samples than had been previously thought. It is also the first demonstration of ED and XRD measured from the same crystal volume enabling direct comparison of X-ray and electron derived data. Ultimately, the work will inform the design and use of high energy (MeV) ED instruments such as HeXI and how those can be complemented by XRD derived information from beamlines such as VMXm.
